# A Mapping Study of Veterinary Literature on Perceptions and Attitudes of Female Canine Spaying

**DOI:** 10.3389/fvets.2020.559659

**Published:** 2020-12-04

**Authors:** Erik Davis Fausak

**Affiliations:** University Library, University of California, Davis, Davis, CA, United States

**Keywords:** perspectives, literature, bitches, dogs, canine, female neuter, spay, ovariectomy

## Abstract

This is a mapping study conducted to evaluate the characteristics of where content that engages in perspectives or attitudes on female dog spaying is published. Three databases, CAB Direct, PubMed, and Scopus, were systematically searched. There were 84 out of 642 papers identified and screened for relevance on attitudes or perceptions on female canine spaying. These 84 articles were then examined for recurring authors, institutional representation, and publisher information. Additionally, information regarding the population being addressed, veterinarian or client, was noted with most literature addressing the veterinary perspective. Many important articles were published in a wide array of journals from many countries, which suggests the importance of not only browsing journals but also searching for relevant literature in databases like CAB Abstracts and MEDLINE.

## Introduction

A great deal of literature and evidence has matured the veterinary approach and perspective toward spaying and neutering, as evidenced by *Frontiers in Veterinary Science* Research Topic: *Effective Options Regarding Spay or Neuter of Dogs* (1). Changing perspectives and risks associated with spaying female dogs can be found in databases as early as 1974 (2). This last decade has seen an explosion in literature regarding perspectives and attitudes in spaying female dogs, particularly an increase in either survey or ethnographic perspectives of specific populations regarding female canine spaying practices (3–13). Additionally, closer examination of a range of disease conditions has potentially been associated with early female spaying (14–29). Even guidelines and evidence-based systematic reviews as well as critically appraised topics have also been published over the past decade (30–36).

This mapping study has been used to identify the trend in literature questioning or examining the convention of spaying female dogs. The growth of evidence-based veterinary medicine has shown that many influential articles may exist outside the scope of typically browsed journal titles (37). This mapping study uses a similar systematic searching approach used in systematic reviews to make reproducible results. The literature retrieved was evaluated by identifying which journals, authors, institutions, countries, and timelines impacted attitudes and perspectives of female canine spaying over the history of veterinary medical literature.

## Methods

The goal of this mapping study was to create a transparent search strategy to identify characteristics of literature regarding female dog neutering in terms of client and veterinary perceptions, including associated risks. The primary approach to this mapping study is based on the concept set forth by Cooper (38). Modification includes conducting a systematized search for literature across three databases (CAB Direct, PubMed, and Scopus) on *female dog spaying*. Selection of which databases to search on female dog neutering was based on a review of database coverage of veterinary literature by Grindlay et al. (39). Specific database search strategies are available (see Appendix A), which included the use of terms for dogs (dog OR dogs OR canines OR canine OR canids OR beagles OR shepherds OR retrievers), spaying (spay^*^ OR OHE OR ovariectomy OR ovariohysterectomy OR “female castration”), and perspectives (Perceptions OR attitudes OR practices OR perception OR ethical OR moral OR “best practices” OR “paradigm” OR evaluation).

Search results were collected and then uploaded into UC Davis Library-licensed *F1000 Workspace* (now *SciWheel*) Citation Management Software and deduplicated. Once deduplicated, a citation management.ris file was exported from *F1000 Workspace* (now *SciWheel*) and uploaded in systematic review software licensed by UC Davis Library, *Covidence* (Australia), under screening and set for only one reviewer (the author). Literature was then screened based on the inclusion/exclusion criteria established. Inclusion criteria included articles in English that included female dogs and spaying and incorporated analysis of cost or benefit, client perspectives, veterinarian perspectives, and addressing potential risks or benefits of spaying. Exclusion occurred with articles not in English, did not involve female dog spaying, focused on procedure (like analgesics or surgical approach), and were case studies.

Once all the articles were screened, they were exported from Covidence and brought into *F1000 Workspace* (now *SciWheel*) for extraction as a.CSV file and analyzed in *Microsoft Excel* (version 16.36).

## Results

There were 642 papers found between the three databases and deduplicated from 722 papers. Only 84 articles were identified to be relevant to perception of spaying and neutering including risk and assessment. Many articles were about procedural refinement including examination of surgical approach and analgesia. Another large group of articles were case reports that included patient signalment in the abstract. CAB Direct had 18% of articles about female dog spaying addressing attitudes and perspectives. PubMed had 13% of articles about female dog spaying addressing attitudes and perspectives. Scopus had 18% of articles about female dog spaying addressing attitudes and perspectives. See Appendix A for summary data of results.

### Journals

**Figure 1:**
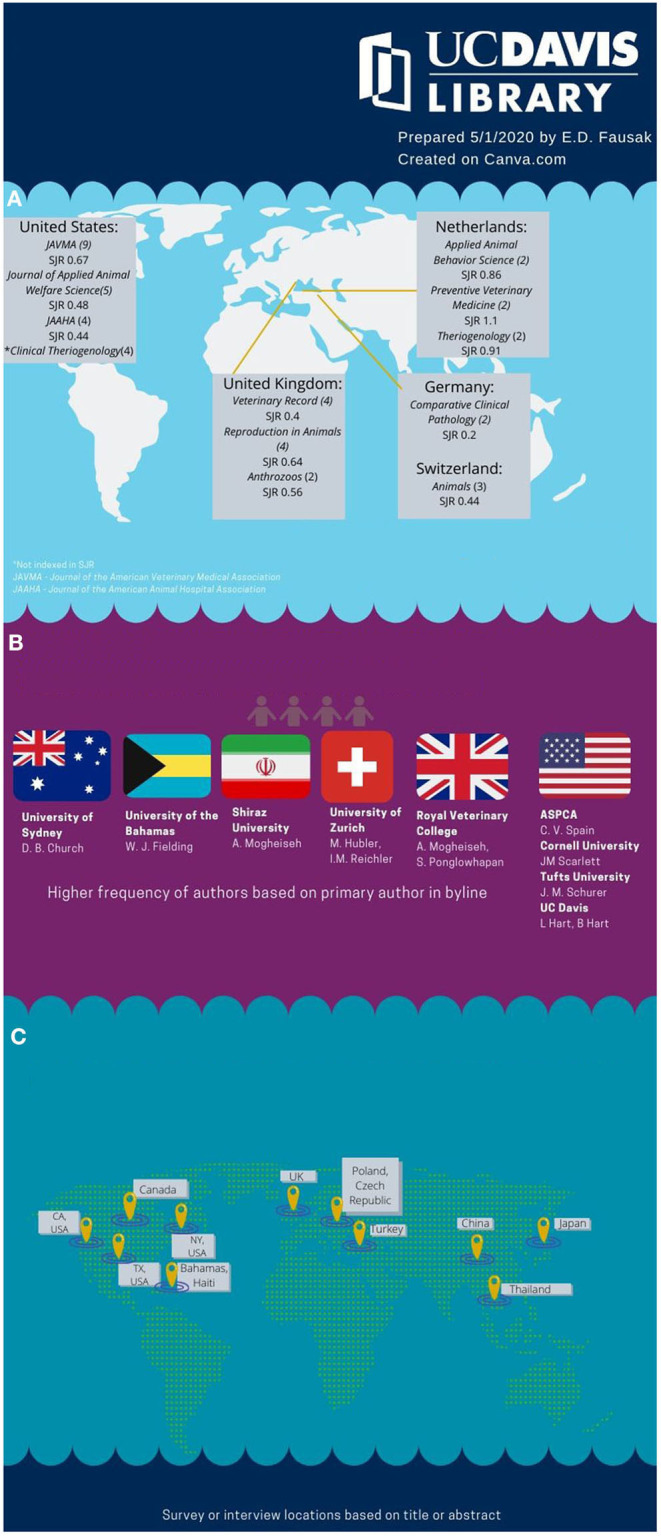
Where perspectives on female dog neutering content is being published. **(A)** Journal (number of articles) and Scimago Journal Ranking (SJR). **(B)** WHO and WHAT Institutions are publishing on perspectives in female dog spaying. **(C)** Parts of the world surveyed or interviewed.

The first article retrieved in the databases that provides perspective on spaying and neutering programs was in *Vet Record* in 1974. Six journals had four or more articles pertaining to perceptions of spaying including the *Journal of the American Veterinary Medical Association* (14, 18, 35, 36, 40–45), the *Journal of Applied Animal Welfare Science* (4, 5, 46–48), *Veterinary Record* (2, 49–51), *Reproduction in Domestic Animals* (29, 52–54), and *Clinical Theriogenology* (22, 26, 55, 56). All journals that had more than one article on the topic are listed with country of origin and Scimago Journal and Country Ranking in Figure 1.

### Authorship and Institutional Contribution

**Figure 2:**
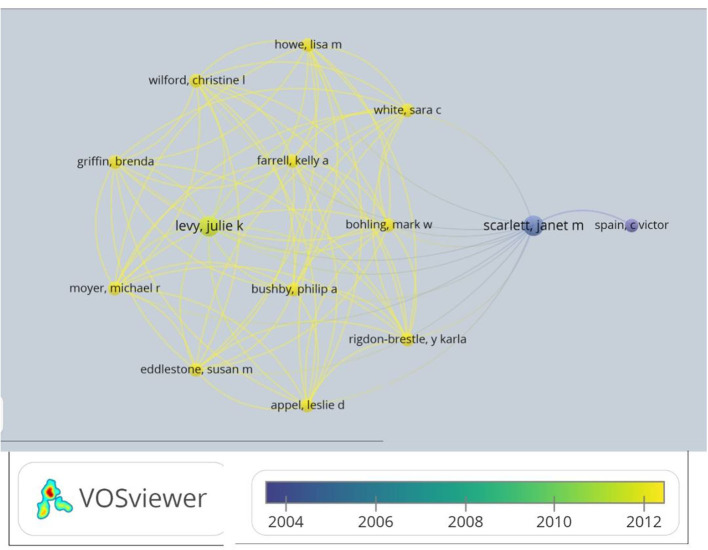
Network analysis of authors. Analysis of authors with more than two publications and their relationship to each other. Built on VOS Viewer. Created by Nees Jan van Eck and Ludo Waltman at Leiden University—May 1, 2020.

Higher frequency of authors (more than one citation as primary name in by-line) that publish articles on perceptions and attitudes toward spaying include Church (University of Sydney, Australia) (17, 57), Fielding (University of Bahamas, Bahamas) (4, 5), Hart (University of California, Davis, USA) (22, 40, 58), Hart (University of California, Davis, USA) (22, 40, 58), Hubler (University of Zurich, Switzerland) (52, 59), Khalid (Royal Veterinary College, United Kingdom) (17, 57), Mogheiseh (Shiraz University, Iran) (60, 61), Ponglowhapan (Royal Veterinary College, United Kingdom) (17, 57), Reichler (University of Zurich, Switzerland) (29, 52, 59), Scarlett (Cornell University, USA) (36, 41, 62), Schurer (Tufts University, USA) (8, 9), and Spain (ASPCA, USA) (41, 62–65). See Figure 1 for author affiliations. Additionally, Figure 2 is a network map of authors by time created in software, VOS Viewer, created by Nees Jan van Eck and Ludo Waltman at Leiden University. Of 286 authors, 22 authors have at least two publications and 14 authors have clear network relationships starting with C. V. Spain of the ASPCA in 2004.

### Surveyed Countries

The US was the most frequently surveyed country with Texas, California, and New York as the most frequently surveyed states (46, 48, 62, 66–68). Multiple surveys were conducted in the United Kingdom, Canada, and Japan (8, 9, 14, 58, 63, 69, 70) (see Figure 1).

### Perspective and Dates of Publication

Most of the articles were written from the perspective of the veterinarian (85%). Many of the articles that address client perspectives utilized surveys or ethnographic data.

The most number of activities of publication on spaying perspectives have been over the last decade with 58 articles since 2010 (3–7, 9, 11–20, 22–29, 31, 35, 44, 48, 52, 54–56, 60, 61, 64, 67, 69, 71–85). The decade of 2000 produced 18 articles (36, 40–43, 46, 47, 49, 50, 57, 59, 62, 63, 66, 71, 86–89). The 1990s produced five articles with only one article published in the 1980s and two in the 1970s (2, 45, 51, 58, 68, 70, 90, 91).

## Conclusions

There were 6,582 articles retrieved with search terms for female dog spaying in English. Of those articles, 642 were retrieved with perceptions or attitudes toward spaying, and 84 articles were screened to pertain to female dog spaying from a client or veterinarian perspective. Of the 642 articles, most were focused on the practice and refinement of spaying as a procedure and were excluded.

Of the top journals, it is probably not surprising that national veterinary organizational journals, like the *Journal of the American Veterinary Medical Association (AVMA)* and *Veterinary Record (BVA)*, are the most common journals to publish in when addressing changing perspectives or attitudes toward female dog spaying. Journals that look at reproduction like *Theriogenology, Clinical Theriogenology*, and *Reproduction in Domestic Animals* are also probably not a surprising source of this content. The broader and more holistic journals are of interest, like *Animals, Anthrozoos*, and *The Journal of Applied Animal Welfare Science*. Many journals that had relevant articles may not be regularly browsed by practitioners.

This mapping study's purpose is to identify literature that examines perspectives about spaying and identify where and when the literature is being published. It is beyond the scope of this paper to assess the quality or content of the literature discovered. This last decade has seen a large increase in literature about what age, in relationship to breed, that female dogs should be spayed. Most of this literature is published for the veterinary professional and very little from a consumer perspective.

What may be interesting to note is that a great deal of consumer health resources may or may not reflect the changes in perspective regarding spaying and neutering. For instance, a gold standard in client information resources, VIN's Veterinary Partner, makes no mention of recent literature on breed-specific concerns in spaying or neutering (92, 93). What is interesting is that most canine food companies like *Hill's Science Diet* or *Royal Canin* do recognize variations in spaying and neutering needs for different breeds (94, 95).

## Data Availability Statement

The original contributions presented in the study are included in the article/supplementary materials, further inquiries can be directed to the corresponding author/s.

## Author Contributions

The author confirms being the sole contributor of this work and has approved it for publication.

## Conflict of Interest

The author declares that the research was conducted in the absence of any commercial or financial relationships that could be construed as a potential conflict of interest. The handling editor declared a shared affiliation with the author at time of review.

## References

[1] Effective Options Regarding Spay or Neuter of Dogs | Frontiers Research Topic (2020). Available online at: https://www.frontiersin.org/research-topics/8524/effective-options-regarding-spay-or-neuter-of-dogs#articles (accessed April 30, 2020).

[2] Joshua JO. Letter: considerations in spaying. Vet Rec. (1974) 94:403–4. 10.1136/vr.94.17.403 4859385

[3] Cocia RI Rusu AS Attitudes of Romanian pet caretakers towards sterilization of their animals: gender conflict over male, but not female, companion animals. Anthrozoös. (2010) 23:185–91. 10.2752/175303710X12682332910097

[4] Fielding WJ. Changing attitudes and animal welfare in small island developing states: dogs on New Providence, The Bahamas. J Appl Anim Welf Sci. (2017) 20:65–74. 10.1080/10888705.2016.1240043 27779423

[5] Fielding WJ Gall M Green D Eller WS. Care of dogs and attitudes of dog owners in Port-au-Prince, the Republic of Haiti. J Appl Anim Welf Sci. (2012) 15:236–53. 10.1080/10888705.2012.683760 22742200

[6] Filipenco N Baraitareanu S Assessment of owner's perception concerning role of neutering and spaying in welfare of dogs. Vet Med. (2012) 58:277–84.

[7] Sontas BH Kaysigiz F Ekici H Methods of oestrus prevention in dogs and cats: a survey of Turkish veterinarian's practices and beliefs. Arch med vet. (2012) 44:155–66. 10.4067/S0301-732X2012000200009

[8] Schurer JM McKenzie C Okemow C Viveros-Guzmán A Beatch H Jenkins EJ. Who let the dogs out? Communicating first nations perspectives on a canine veterinary intervention through digital storytelling. Ecohealth. (2015) 12:592–601. 10.1007/s10393-015-1055-y 26302958

[9] Schurer JM Phipps K Okemow C Beatch H Jenkins E. Stabilizing dog populations and improving animal and public health through a participatory approach in indigenous communities. Zoonoses Public Health. (2015) 62:445–55. 10.1111/zph.12173 25439233

[10] Toukhsati SR Phillips CJC Podberscek AL Coleman GJ Companion animals in Thailand. Soc Anim. (2015) 23:569–93. 10.1163/15685306-12341381

[11] Valenta K Gettinger-Larson JA Chapman CA Farris ZJ Barking up the right tree: understanding local attitudes towards dogs in villages surrounding Ranomafana National Park, Madagascar can benefit applied conservation. Madagascar Conserv Dev. (2016) 11:87 10.4314/mcd.v11i2.4

[12] Dias Costa E Martins CM Cunha GR Catapan DC Ferreira F Oliveira ST. Impact of a 3-year pet management program on pet population and owner's perception. Prev Vet Med. (2017) 139(Pt. A):33–41. 10.1016/j.prevetmed.2017.01.001 28364830

[13] Hsueh C Giuffrida M Mayhew PD Case JB Singh A Monnet E. Evaluation of pet owner preferences for operative sterilization techniques in female dogs within the veterinary community. Vet Surg. (2018) 47:O15–25. 10.1111/vsu.12766 29400403

[14] McIntyre RL Levy JK Roberts JF Reep RL. Developmental uterine anomalies in cats and dogs undergoing elective ovariohysterectomy. J Am Vet Med Assoc. (2010) 237:542–6. 10.2460/javma.237.5.542 20807131

[15] McKenzie B Evaluating the benefits and risks of neutering dogs and cats. CAB Rev. (2010) 5:1–18. 10.1079/PAVSNNR20105045

[16] de Bleser B Brodbelt DC Gregory NG Martinez TA. The association between acquired urinary sphincter mechanism incompetence in bitches and early spaying: a case-control study. Vet J. (2011) 187:42–7. 10.1016/j.tvjl.2009.11.004 20004121

[17] Ponglowhapan S Khalid M Church D Canine urinary incontinence post-neutering: a review of associated factors, pathophysiology and treatment options. Thai J Vet Med. (2012) 42:259–65.

[18] Forsee KM Davis GJ Mouat EE Salmeri KR Bastian RP. Evaluation of the prevalence of urinary incontinence in spayed female dogs: 566 cases (2003-2008). J Am Vet Med Assoc. (2013) 242:959–62. 10.2460/javma.242.7.959 23517208

[19] Nolen RS. Study shines spotlight on neutering: assumptions about a mainstay of companion animal practice are called into question. J Am Vet Med Assoc. (2013) 243:1218–23. 10.2460/javma.243.9.1218 24134569

[20] Pak SI A cross-sectional study on the prevalence of canine obesity and associated risk factors in Chuncheon, Kangwon province. J Vet Clin. (2014) 31:31–5. 10.17555/ksvc.2014.02.31.1.31

[21] Howe L. Current perspectives on the optimal age to spay/castrate dogs and cats. VMRR. (2015) 6:171–80. 10.2147/VMRR.S53264 30101104 PMC6070019

[22] Hart B Hart L Thigpen A Willits N Best age for spay and neuter: a new paradigm. Clin Theriogenol. (2019) 11:235–7.

[23] Starling M Fawcett A Wilson B Serpell J McGreevy P. Behavioural risks in female dogs with minimal lifetime exposure to gonadal hormones. PLoS ONE. (2019) 14:e0223709. 10.1371/journal.pone.0223709 31805064 PMC6894801

[24] Yates D Leedham R Prepubertal neutering in cats and dogs. Clin Pract. (2019) 41:285–98. 10.1136/inp.l5007

[25] Yates D Leedham R Prepubertal neutering of dogs — some risks and benefits. Companion Animal. (2019) 24:38–42. 10.12968/coan.2019.24.1.38

[26] Sones E Neoplastic considerations for spaying and neutering dogs. Clin Theriogenol. (2019) 11:239–42.

[27] Scandurra A Alterisio A Di Cosmo A D'Ambrosio A D'Aniello B. Ovariectomy impairs socio-cognitive functions in dogs. Animals. (2019) 9:58. 10.3390/ani9020058 30769794 PMC6406991

[28] Mongillo P Scandurra A D'Aniello B Marinelli L Effect of sex and gonadectomy on dogs' spatial performance. Appl Anim Behav Sci. (2017) 191:84–9. 10.1016/j.applanim.2017.01.017

[29] Balogh O Borruat N Andrea Meier A Hartnack S Reichler IM. The influence of spaying and its timing relative to the onset of puberty on urinary and general behaviour in labrador retrievers. Reprod Domest Anim. (2018) 53:1184–90. 10.1111/rda.13225 29974985

[30] Mwangi WE Mogoa EM Mwangi JN Mbuthia PG Mbugua SW. A systematic review of analgesia practices in dogs undergoing ovariohysterectomy. Vet World. (2018) 11:1725–35. 10.14202/vetworld.2018.1725-1735 30774265 PMC6362335

[31] D'Onise K Hazel S Caraguel C. Mandatory desexing of dogs: one step in the right direction to reduce the risk of dog bite? a systematic review. Inj Prev. (2017) 23:212–8. 10.1136/injuryprev-2016-042141 28130398

[32] Benavides Melo CJ Astaíza Martínez JM Rojas ML Complications due to surgical sterilization by ovariohysterectomy in female dogs: a systematic review. Rev Med Vet. (2018) 37:83–93. 10.19052/mv.vol1.iss37.10

[33] Beauvais W Cardwell JM Brodbelt DC. The effect of neutering on the risk of urinary incontinence in bitches - a systematic review. J Small Anim Pract. (2012) 53:198–204. 10.1111/j.1748-5827.2011.01176.x 22353203

[34] Stavisky J Wareham K Age at Neutering and Mammary Tumours in Bitches. BestBETs for Vets (2018). Available online at: https://bestbetsforvets.org/bet/144 (accessed April 30, 2020).

[35] Association of Shelter Veterinarians' Veterinary Task Force to Advance Spay-Neuter Griffin B Bushby PA McCobb E White SC Rigdon-Brestle YK. The association of shelter veterinarians' 2016 veterinary medical care guidelines for spay-neuter programs. J Am Vet Med Assoc. (2016) 249:165–88. 10.2460/javma.249.2.165 27379593

[36] Looney AL Bohling MW Bushby PA Howe LM Griffin B Levy JK. The association of shelter veterinarians veterinary medical care guidelines for spay-neuter programs. J Am Vet Med Assoc. (2008) 233:74–86. 10.2460/javma.233.1.74 18593314

[37] Williams HC. Evidence-based veterinary dermatology–better to light a candle than curse the darkness. Vet Dermatol. (2010) 21:1–3. 10.1111/j.1365-3164.2009.00873.x 20187909

[38] Cooper ID What is a “mapping study?” J Med Libr Assoc. (2016) 104:76–8. 10.3163/1536-5050.104.1.013 26807058 PMC4722648

[39] Grindlay DJC Brennan ML Dean RS. Searching the veterinary literature: a comparison of the coverage of veterinary journals by nine bibliographic databases. J Vet Med Educ. (2012) 39:404–12. 10.3138/jvme.1111.109R 23187034

[40] Hart BL. Effect of gonadectomy on subsequent development of age-related cognitive impairment in dogs. J Am Vet Med Assoc. (2001) 219:51–6. 10.2460/javma.2001.219.51 11439769

[41] Spain CV Scarlett JM Houpt KA. Long-term risks and benefits of early-age gonadectomy in dogs. J Am Vet Med Assoc. (2004) 224:380–7. 10.2460/javma.2004.224.380 14765797

[42] Feeser P. Thoughts on spay-neuter program guidelines. J Am Vet Med Assoc. (2008) 233:1056–7. 10.2460/javma.233.7.1053 18839490

[43] Milani M. Eucourages discussion on spay and neuter of dogs and cats. J Am Vet Med Assoc. (2008) 232:194. 10.2460/javma.232.2.194 18290266

[44] Zink MC Farhoody P Elser SE Ruffini LD Gibbons TA Rieger RH Evaluation of the risk and age of onset of cancer and behavioral disorders in gonadectomized Vizslas. J Am Vet Med Assoc. (2014) 244:309–19. 10.2460/javma.244.3.309 24432963

[45] Cloud DF. Working with breeders on solutions to pet overpopulation. J Am Vet Med Assoc. (1993) 202:912–4. 8468213

[46] Faver CA. Sterilization of companion animals: exploring the attitudes and behaviors of Latino students in south Texas. J Appl Anim Welf Sci. (2009) 12:314–30. 10.1080/10888700903163534 20183484

[47] McKay SA Farnworth MJ Waran NK. Current attitudes toward, and incidence of, sterilization of cats and dogs by caregivers (owners) in Auckland, New Zealand. J Appl Anim Welf Sci. (2009) 12:331–44. 10.1080/10888700903163617 20183485

[48] Dolan ED Weiss E Slater MR. Welfare impacts of spay/neuter-focused outreach on companion animals in New York City public housing. J Appl Anim Welf Sci. (2017) 20:257–72. 10.1080/10888705.2017.1305904 28481141

[49] When should bitches be neutered Vet Rec. (2001) 148:491–3. 11345988

[50] Tivers M Travis T Windsor R. Survey of neutering practices. Vet Rec. (2000) 147:667. 11131557

[51] Thornton PD. Early neutering of cats and dogs. Vet Rec. (1998) 142:200. 9533287

[52] Reichler IM Hubler M. Urinary incontinence in the bitch: an update. Reprod Domest Anim. (2014) 49(Suppl. 2):75–80. 10.1111/rda.12298 24947864

[53] Szczubial M Kankofer M Bochniarz M Dabrowski R. Effects of ovariohysterectomy on oxidative stress markers in female dogs. Reprod Domest Anim. (2015) 50:393–9. 10.1111/rda.12501 25704084

[54] Hagman R. Canine pyometra: what is new? Reprod Domest Anim. (2017) 52(Suppl. 2):288–92. 10.1111/rda.12843 27807901

[55] Bailey CS Non-cancerous conditions associated with spay/neuter status in the canine. Clin Theriogenol. (2016) 8:203–6.

[56] Brent L Growing interest in hormone sparing dog sterilization and recommendations for standard identification methods. Clin Theriogenol. (2019) 11:247–53.

[57] Ponglowhapan S Church DB Khalid M. Differences in the proportion of collagen and muscle in the canine lower urinary tract with regard to gonadal status and gender. Theriogenology. (2008) 70:1516–24. 10.1016/j.theriogenology.2008.06.099 18703223

[58] Hart LA Takayanagi T Yamaguchi C Dogs and cats in animal shelters in japan. Anthrozoös. (1998) 11:157–63. 10.2752/089279398787000706

[59] Reichler IM Welle M Sattler U Jöchle W Roos M Hubler M. Comparative quantitative assessment of GnRH- and LH-receptor mRNA expression in the urinary tract of sexually intact and spayed female dogs. Theriogenology. (2007) 67:1134–42. 10.1016/j.theriogenology.2007.01.001 17276503

[60] Mogheiseh A Nikahval B Ahrari Khafi MS Mansourian M Nazifi S Mardani Z Effects of bilateral whole vessel ovarian ligation on dogs' ovarian function and histopathology. Comp Clin Path. (2018) 27:1085–91. 10.1007/s00580-018-2705-6

[61] Mogheiseh A Nikahval B Ahmadi N Yazdanpanah R Sadat Z Nazifi S Bilateral ovarian pedicle ligation as an alternative to ovariectomy and ovarian response to eCG treatment. Comp Clin Path. (2017) 26:197–202. 10.1007/s00580-016-2369-z

[62] Spain CV Scarlett JM Cully SM When to neuter dogs and cats: a survey of New York state veterinarians' practices and beliefs. J Am Anim Hosp Assoc. (2002) 38:482–8. 10.5326/0380482 12220034

[63] Houpt KA Goodwin D Uchida Y Baranyiová E Fatjó J Kakuma Y Proceedings of a workshop to identify dog welfare issues in the US, Japan, Czech Republic, Spain and the UK. Appl Anim Behav Sci. (2007) 106:221–33. 10.1016/j.applanim.2007.01.005

[64] Perez-Marin CC Molina L Vizuete G Sanchez JM Zafra R Bautista MJ Uterine and ovarian remnants in an incorrectly spayed bitch: a case report. Vet Med. (2014) 59:102–6. 10.17221/7320-VETMED

[65] Villaverde Haro C Canine and Feline Obesity - Tackling a Growing Problem. Vet Times. (2015).

[66] Snowden K Bice K Craig T Howe L Jarrett M Jeter E. Vertically integrated educational collaboration between a college of veterinary medicine and a non-profit animal shelter. J Vet Med Educ. (2008) 35:637–40. 10.3138/jvme.35.4.637 19228920

[67] Kass PH Johnson KL Weng H-Y. Evaluation of animal control measures on pet demographics in Santa Clara County, California, 1993-2006. PeerJ. (2013) 1:e18. 10.7717/peerj.18 23638352 PMC3628371

[68] Vasseur PB Berger B Leighton RL The volume and distribution of surgical cases in 78 small animal practices in California. J Am Anim Hosp Assoc. (1981) 17:161–6. 10.1111/j.1752-1688.1981.tb02612.x

[69] Adams V Walker S Taylor C Attitudes to and opinions of neutering in dogs: results of a canine reproduction survey of veterinary surgeons. In: BSAVA Congress Proceedings. British Small Animal Veterinary Association (2016) p. 475–6. 10.22233/9781910443446.54.12

[70] Thrusfield MV Holt PE Muirhead RH. Acquired urinary incontinence in bitches: its incidence and relationship to neutering practices. J Small Anim Pract. (1998) 39:559–66. 10.1111/j.1748-5827.1998.tb03709.x 9888109

[71] Byron JK Graves TK Becker MD Cosman JF Long EM. Evaluation of the ratio of collagen type III to collagen type I in periurethral tissues of sexually intact and neutered female dogs. Am J Vet Res. (2010) 71:697–700. 10.2460/ajvr.71.6.697 20513187

[72] Gunay A Gunes N Gunay U Effect of ovariohysterectomy on lipid peroxidation and levels of some antioxidants and biochemical parameters in bitches. Bull Vet Inst Puławy. (2011) 55:695–8.

[73] Miles CR Bell CM Pinkerton ME Soukup JW. Maxillary ameloblastic fibroma in a dog. Vet Pathol. (2011) 48:823–6. 10.1177/0300985810382091 20861502

[74] Bushby PA Griffin B An overview of pediatric spay and neuter benefits and techniques. Vet Med. (2011) 106:83–9.

[75] Zanowski GN. A fresh look at spay/neuter legislation: the journey to a middle ground. J Public Health Manag Pract. (2012) 18:E24–33. 10.1097/PHH.0b013e318222a7f5 22473127

[76] Muraro L White RS. Complications of ovariohysterectomy procedures performed in 1880 dogs. Tierarztl Prax Ausg K Kleintiere Heimtiere. (2014) 42:297–302. 10.1055/s-0038-1623776 25323211

[77] Bednarczyk-Szurmak M Bombik E Bombik T Łagowska K Szumigłowska I Rózewicz M Fighting homelessness of dogs - evaluation of the strategy adapted by the city of Siedlce. Acta Scientiarum Polonorum - Zootechnica. (2015) 14:25–32.

[78] Selting KA Relationship between neuter status and cancer highlighted by global differences in neutering practices. In: 40th World Small Animal Veterinary Association Congress. Bangkok (2015). p. 883–4.

[79] Lima de LR Fontana CD Eda HM Ribeiro RM Faleiros RR Low-cost neutering program and its postsurgical complications for dogs and cats. Int J Vet Sci. (2016) 5:122–6.

[80] Jupe A Rand J Morton J Fleming S. Attitudes of veterinary teaching staff and exposure of veterinary students to early-age desexing, with review of current early-age desexing literature. Animals. (2017) 8:3. 10.3390/ani8010003 29295577 PMC5789298

[81] Palmer C Pedersen HG Sandøe P Beyond castration and culling: should we use non-surgical, pharmacological methods to control the sexual behavior and reproduction of animals? J Agric Environ Ethics. (2018) 31:197–218. 10.1007/s10806-018-9718-7

[82] Garcia RCM Amaku M Biondo AW Ferreira F Dog and cat population dynamics in an urban area: evaluation of a birth control strategy. Pesq Vet Bras. (2018) 38:511–8. 10.1590/1678-5150-pvb-4205

[83] Hoad JG Spaying bitches: why, when, how? Vet Nurs. (2018) 9:418–21. 10.12968/vetn.2018.9.8.418

[84] Urfer SR Kaeberlein M. Desexing dogs: a review of the current literature. Animals. (2019) 9:1086. 10.3390/ani9121086 31817504 PMC6940997

[85] Bjørnvad CR Gloor S Johansen SS Sandøe P Lund TB. Neutering increases the risk of obesity in male dogs but not in bitches - a cross-sectional study of dog- and owner-related risk factors for obesity in Danish companion dogs. Prev Vet Med. (2019) 170:104730. 10.1016/j.prevetmed.2019.104730 31421500

[86] Slauterbeck JR Pankratz K Xu KT Bozeman SC Hardy DM. Canine ovariohysterectomy and orchiectomy increases the prevalence of ACL injury. Clin Orthop Relat Res. (2004) 429:301–5. 10.1097/01.blo.0000146469.08655.e2 15577502

[87] Opperman M Cultivate a welcoming practice. Vet Econ. (2005) 46:52–8.

[88] Risley-Curtiss C Holley LC Wolf S. The animal-human bond and ethnic diversity. Soc Work. (2006) 51:257–68. 10.1093/sw/51.3.257 17076123

[89] Pelander L Hagman R Häggström J. Concentrations of cardiac Troponin I before and after ovariohysterectomy in 46 female dogs with pyometra. Acta Vet Scand. (2008) 50:35. 10.1186/1751-0147-50-35 18786242 PMC2546406

[90] Wildt DE Kinney GM Seager SWJ Reproduction control in the dog and cat: an examination and evaluation of current and proposed methods. J Am Anim Hosp Assoc. (1977) 13:223–31.

[91] Rollin BE. An ethicist's commentary on the case of the pregnant dog brought in for a spay and found to be pregnant. Can Vet J. (1998) 39:399. 9759507 PMC1539532

[92] Brooks W Spaying your female dog. Vet Part. (2018). Available online at: https://veterinarypartner.vin.com/default.aspx?pid=19239&id=4951464

[93] Kogan LR Schoenfeld-Tacher R Gould L Viera AR Hellyer PW. Providing an information prescription in veterinary medical clinics: a pilot study. J Med Libr Assoc. (2014) 102:41–6. 10.3163/1536-5050.102.1.008 24415918 PMC3878934

[94] When to Spay a Female Dog? - Royal Canin (2020). Available online at: https://www.royalcanin.com/us/dogs/puppy/when-to-spay-a-female-dog (accessed December 30, 2019).

[95] When Should a Dog Be Spayed? | Hill's Pet. (2020). Available online at: https://www.hillspet.com/dog-care/healthcare/when-to-spay-female-dogs (accessed December 30, 2019).

