# Supplementary material for: A Mapping Study of Veterinary Literature on Perceptions and Attitudes of Female Canine Spaying
Source: Front Vet Sci. 2020 Dec 4;7:559659. doi: 10.3389/fvets.2020.559659 (PMC7746841; doi:10.3389/fvets.2020.559659)
Supplement: Supplementary file 1 [file Data_Sheet_1.pdf]

Appendix A: search summary and result data

A Mapping Study of Veterinary Literature on Perceptions and Attitudes of Female Canine Ovariohysterectomies. Erik Davis Fausak.

Table A: Databases searched

**Database**	**Interface**	**Date Coverage**	**Date Searched(d.m.yr)**
CAB ABstracts	CABDirect	1910 to Present	27.12.2019
Medline	PubMed	1946-Present	27.12.2019.
Scopus	Scopus	1823-Present	27.12.2019

Table B: CAB Direct Search Strategy

**Search ID**	**Terms (copy and paste)**	**Results**
**#1 Spaying**	**ti:(spay* OR OHE OR ovariectomy OR ovariohysterectomy OR "female castration") OR ab:(spay* OR OHE OR ovariectomy OR ovariohysterectomy OR "female castration") OR de:(ovariectomy)**	**13,183**
**#2 Canines**	**ti:(dog OR dogs OR canines OR canine OR canids OR beagles OR shepherds OR retrievers) OR ab:(dog OR dogs OR canines OR canine OR canids OR beagles OR shepherds OR retrievers) OR OD:(dogs)**	**230,401**
**#3 Perceptions**	**Ti: (Perceptions OR attitudes OR practices OR perception OR ethical OR moral OR “best practices” OR “paradigm” OR evaluation) OR ab:(Perceptions OR attitudes OR practices OR perception OR ethical OR moral OR “best practices” OR “paradigm” OR evaluation) OR de:(“risk assessment”)**	**601.366**

**#4**	**#1 AND #2 AND #3**	**564**
**#5**	**#4 AND SC:VE**	**562**
**#6 LIterature that focuses on female neutering perspectives**	**#5 AND Language: English**	**478**
**#7**	**#1 AND #2**	**3,354**
**#8**	**#7 AND sc:VE**	**3,338**
**#9 Literature on female neutering in general**	**#8 AND Lang:Eng**	**2,703**

Table C: PubMed Search Strategy

**Search ID**	**Terms (copy and paste)**	**Results**
**#1 Spaying**	**"ovariectomy"[MeSH Terms] OR "ovariectomy"[tiab] OR “spay”[tiab] OR “spaying”[tiab] OR “ovariohysterectomy”[tiab] OR “female castration”[tiab]**	**29,987**
**#2 Canine**	**"dogs"[MeSH Terms] OR “canis”[tiab] OR “dog”[tiab] OR “dogs”[tiab] OR “canines”[tiab] OR “canine”[tiab] OR “canids”[tiab] OR “beagles”[tiab] OR “shepherds”[tiab] OR “retrievers”[tiab]**	**373,345**
**#3 Perceptions**	**"perception"[MeSH Terms] OR "attitude"[MeSH Terms] OR "perception"[MeSH Terms] OR "morals"[MeSH Terms] OR “Perceptions”[tiab] OR “attitudes”[tiab] OR “practices”[tiab] OR “perception”[tiab] OR “ethical”[tiab] OR “moral”[tiab] OR “best practices”[tiab] OR “paradigm”[tiab] OR “evaluation”[tiab]**	**2,609,843**
**#4**	**#1 AND #2 AND #3**	**168**
**#5 LIterature that focuses**	**#4 AND English**	**162**

**on female neutering perspectives**		
**#6 Literature on female neutering in general**	**#1 AND #2 AND English**	**1,261**

Table D: Scopus Search Strategy

**Search ID**	**Terms (copy and paste)**	**Results**
**#1 Spaying**	TITLE-ABS-KEY (spay* OR ohe OR ovariectomy OR ovariohysterectomy OR "female castration")	**47,611**
**#2 Canine**	TITLE-ABS-KEY (dog OR dogs OR canines OR canine OR canids OR beagles OR shepherds OR retrievers OR canis)	**486,057**
**#3 Perceptions**	TITLE-ABS-KEY (perceptions OR attitudes OR practices OR perception OR ethical OR moral OR "best practices" OR "paradigm" OR evaluation)	**7,626,208**
**#4**	#1 AND #2 AND #3	**514**
**#5 LIterature that focuses on female neutering perspectives**	#4 AND English	**475**
**#6 Literature on female neutering in general**	#1 AND #2 AND English	**2,618**

Table E: Inclusion/Exclusion criteria for Covidence

Inclusion	Exclusion
**English**<br>**Female Dogs**<br>**Analysis of cost and benefit to female spaying**<br>**Addresses client perspectives about spaying**<br>**Addresses veterinarian perspectives about spaying**<br>**Addresses potential risks or benefits of spaying**	**Not in English**<br>**Not female dogs**<br>**Procedural evaluation (like choice and evaluation of analgesics or surgical approach)**<br>**Not about spaying female dogs**<br>**A Case Study (that only includes signalment or unique spaying instance)**

Table F: Results

Database	Total Records	Total Records after deduplication	Deduplication software/methodology
	722	642	F1000

Table G: Journals with more than one citation.

Journal	# articles	Country of Origin	SJR Score
Journal of the American Veterinary Medical Association	**9**	**USA**	**0.67**
Journal of Applied Animal Welfare Science	**5**	**USA**	**0.48**
Veterinary Record	**4**	**United Kingdom**	**0.4**
Reproduction in Domestic Animals	**4**	**United Kingdom**	**0.64**

Journal of the American Animal Hospital Association	4	USA	0.44
Clinical Theriogenology	4	USA	Not Indexed
Animals	3	Switzerland	0.67
Anthrozoos	2	United Kingdom	0.56
Applied Animal Behaviour Science	2	Netherlands	0.86
Comparative Clinical Pathology	2	Germany	0.2
Preventive Veterinary Medicine	2	Netherlands	1.1
Theriogenology	2	Netherlands	0.91

Table H: Frequently represented authors (primary by-line) and institutions

Author	Institution
D. B. Church	University of Sydney, Australia
W. J. Fielding	University of the Bahamas, Bahamas
B. Hart	University of California, Davis, USA
L. Hart	University of California, Davis, USA
M. Hubler	University of Zurich, Switzerland

**M. Khalid**	**Royal Veterinary College, United Kingdom**
**A. Mogheiseh**	**Shiraz University, Iran**
**S. Ponglowhapan**	**Royal Veterinary College, United Kingdom**
**I. M. Reichler**	**University of Zurich, Switzerland**
**J. M. Scarlett**	**Cornell University, USA**
**J. M. Schurer**	**Tufts University, USA**
**C. V. Spain**	**ASPCA, USA**

Table I: Surveyed Countries (veterinarian or client surveys)

Country	Survey Articles
USA	13
Japan	2
Canada	3
UK	5
Czech Republic	1
Spain	1
New Zealand	1
Haiti	1
Turkey	1
Poland	1
China	1

**Thailand**	**1**
**Bahamas**	**1**

Table J: Number of articles on canine female spaying in five year increments

**Years Published**	**Number of Articles**
**2015-2020**	**37**
**2010-2015**	**21**
**2005-2010**	**12**
**2000-2005**	**6**
**1995-2000**	**4**
**Pre-1995**	**4**

84 References included after first screen of title and abstracts in Covidence

1.
Hart LA, Takayanagi T, Yamaguchi C. Dogs and cats in animal shelters in japan. *Anthrozoös* (1998) 11:157–163. doi:10.2752/089279398787000706
2.
Spain CV, Scarlett JM, Cully SM. When to neuter dogs and cats: a survey of New York state veterinarians' practices and beliefs. *J Am Anim Hosp Assoc* (2002) 38:482–488. doi:10.5326/0380482
3.
Slauterbeck JR, Pankratz K, Xu KT, Bozeman SC, Hardy DM. Canine ovariohysterectomy and orchiectomy increases the prevalence of ACL injury. *Clin Orthop Relat Res* (2004)301–305. doi:10.1097/01.blo.0000146469.08655.e2
4.
Opperman M. Cultivate a welcoming practice. *Vet Econ* (2005) 46:52–58.
5.
Risley-Curtiss C, Holley LC, Wolf S. The animal-human bond and ethnic diversity. *Soc Work* (2006) 51:257–268. doi:10.1093/sw/51.3.257
6.
Zanowski GN. A fresh look at spay/neuter legislation: the journey to a middle ground. *J Public Health Manag Pract* (2012) 18:E24-33. doi:10.1097/PHH.0b013e318222a7f5
7.
Sontas BH, Kaysigiz F, Ekici H. Methods of oestrus prevention in dogs and cats: a survey of Turkish veterinarian's practices and beliefs. *Arch med vet* (2012) 44:155–166. doi:10.4067/S0301-732X2012000200009
8.
Ponglowhapan S, Khalid M, Church D. Canine urinary incontinence post-neutering: A review of associated factors, pathophysiology and treatment options. *Thai J Vet Med* (2012) 42:259–265.
9.

Nolen RS. Study shines spotlight on neutering: assumptions about a mainstay of companion animal practice are called into question. *J Am Vet Med Assoc* (2013) 243:1218–1223.
10.
Pak SI. A cross-sectional study on the prevalence of canine obesity and associated risk factors in Chuncheon, Kangwon province. *J Vet Clin* (2014) 31:31–35.
11.
Toukhsati SR, Phillips CJC, Podberscek AL, Coleman GJ. Companion animals in thailand. *Soc Animals* (2015) 23:569–593. doi:10.1163/15685306-12341381
12.
Schurer JM, Phipps K, Okemow C, Beatch H, Jenkins E. Stabilizing dog populations and improving animal and public health through a participatory approach in indigenous communities. *Zoonoses Public Health* (2015) 62:445–455. doi:10.1111/zph.12173
13.
Schurer JM, McKenzie C, Okemow C, Viveros-Guzmán A, Beatch H, Jenkins EJ. Who let the dogs out? communicating first nations perspectives on a canine veterinary intervention through digital storytelling. *Ecohealth* (2015) 12:592–601. doi:10.1007/s10393-015-1055-y
14.
Hagman R. Canine pyometra: What is new? *Reprod Domest Anim* (2017) 52 Suppl 2:288–292. doi:10.1111/rda.12843
15.
Starling M, Fawcett A, Wilson B, Serpell J, McGreevy P. Behavioural risks in female dogs with minimal lifetime exposure to gonadal hormones. *PLoS ONE* (2019) 14:e0223709. doi:10.1371/journal.pone.0223709
16.
Yates D, Leedham R. Prepubertal neutering in cats and dogs. *In Pract* (2019) 41:285–298. doi:10.1136/inp.l5007
17.
Urfer SR, Kaeberlein M. Desexing dogs: A review of the current literature. *Animals (Basel)* (2019) 9: doi:10.3390/ani9121086
18.
Joshua JO. Letter: Considerations in spaying. *Vet Rec* (1974) 94:403–404. doi:10.1136/vr.94.17.403
19.
Thornton PD. Early neutering of cats and dogs. *Vet Rec* (1998) 142:200.
20.
Rollin BE. An ethicist's commentary on the case of the pregnant dog brought in for a spay and found to be pregnant. *Can Vet J* (1998) 39:399.
21.
Tivers M, Travis T, Windsor R. Survey of neutering practices. *Vet Rec* (2000) 147:667.
22.
When should bitches be neutered. *Vet Rec* (2001) 148:491–493.
23.
Milani M. Eucourages discussion on spay and neuter of dogs and cats. *J Am Vet Med Assoc* (2008) 232:194.
24.
Feeser P. Thoughts on spay-neuter program guidelines. *J Am Vet Med Assoc* (2008) 233:1056; author reply 1056-7.
25.
Byron JK, Graves TK, Becker MD, Cosman JF, Long EM. Evaluation of the ratio of collagen type III to collagen type I in periurethral tissues of sexually intact and neutered female dogs. *Am J Vet Res* (2010) 71:697–700. doi:10.2460/ajvr.71.6.697
26.
Zink MC, Farhoody P, Elser SE, Ruffini LD, Gibbons TA, Rieger RH. Evaluation of the risk and age of onset of cancer and behavioral disorders in gonadectomized Vizslas. *J Am Vet Med Assoc* (2014) 244:309–319. doi:10.2460/javma.244.3.309
27.
Association of Shelter Veterinarians' Veterinary Task Force to Advance Spay-Neuter, Griffin B, Bushby PA, McCobb E, White SC, Rigdon-Brestle YK, Appel LD, Makolinski KV, Wilford CL, Bohling MW, et al. The Association of Shelter Veterinarians' 2016 Veterinary Medical Care Guidelines for Spay-Neuter Programs. *J Am Vet Med Assoc* (2016) 249:165–188. doi:10.2460/javma.249.2.165
28.
Dias Costa E, Martins CM, Cunha GR, Catapan DC, Ferreira F, Oliveira ST, Garcia R de CM, Biondo AW. Impact of a 3-year pet management program on pet population and owner's perception. *Prev Vet Med* (2017) 139:33–41. doi:10.1016/j.prevetmed.2017.01.001
29.

Jupe A, Rand J, Morton J, Fleming S. Attitudes of Veterinary Teaching Staff and Exposure of Veterinary Students to Early-Age Desexing, with Review of Current Early-Age Desexing Literature. *Animals (Basel)* (2017) 8: doi:10.3390/ani8010003
30.
Hsueh C, Giuffrida M, Mayhew PD, Case JB, Singh A, Monnet E, Holt DE, Cray M, Curcillo C, Runge JJ. Evaluation of pet owner preferences for operative sterilization techniques in female dogs within the veterinary community. *Vet Surg* (2018) 47:O15–O25. doi:10.1111/vsu.12766
31.
Bjørnvad CR, Gloor S, Johansen SS, Sandøe P, Lund TB. Neutering increases the risk of obesity in male dogs but not in bitches - A cross-sectional study of dog- and owner-related risk factors for obesity in Danish companion dogs. *Prev Vet Med* (2019) 170:104730. doi:10.1016/j.prevetmed.2019.104730
32.
Wildt DE, Kinney GM, Seager SWJ. Reproduction control in the dog and cat: an examination and evaluation of current and proposed methods. *Journal of the American Animal Hospital Association* (1977) 13:223–231.
33.
Vasseur PB, Berger B, Leighton RL. The volume and distribution of surgical cases in 78 small animal practices in California. *Journal of the American Animal Hospital Association* (1981) 17:161–166.
34.
Thrusfield MV, Holt PE, Muirhead RH. Acquired urinary incontinence in bitches: its incidence and relationship to neutering practices. *J Small Anim Pract* (1998) 39:559–566. doi:10.1111/j.1748-5827.1998.tb03709.x
35.
Selting KA. Relationship between neuter status and cancer highlighted by global differences in neutering practices. (2015)883–884.
36.
Cloud DF. Working with breeders on solutions to pet overpopulation. *Journal of the American Veterinary Medical Association* (1993) 202:912–914.
37.
Reichler IM, Hubler M. Urinary incontinence in the bitch: an update. *Reprod Domest Anim* (2014) 49 Suppl 2:75–80. doi:10.1111/rda.12298
38.
Shmalberg J, Memon MA. A retrospective analysis of 5,195 patient treatment sessions in an integrative veterinary medicine service: patient characteristics, presenting complaints, and therapeutic interventions. *Vet Med Int* (2015) 2015:983621. doi:10.1155/2015/983621
39.
Gunay A, Gunes N, Gunay U. Effect of ovariohysterectomy on lipid peroxidation and levels of some antioxidants and biochemical parameters in bitches. *Bulletin of the Veterinary Institute in Puławy* (2011) 55:695–698.
40.
Snowden K, Bice K, Craig T, Howe L, Jarrett M, Jeter E, Kochevar D, Simpson RB, Stickney M, Wesp A, et al. Vertically integrated educational collaboration between a college of veterinary medicine and a non-profit animal shelter. *J Vet Med Educ* (2008) 35:637–640. doi:10.3138/jvme.35.4.637
41.
Spain CV, Scarlett JM, Houpt KA. Long-term risks and benefits of early-age gonadectomy in dogs. *J Am Vet Med Assoc* (2004) 224:380–387. doi:10.2460/javma.2004.224.380
42.
Palmer C, Pedersen HG, Sandøe P. Beyond Castration and Culling: Should We Use Non-surgical, Pharmacological Methods to Control the Sexual Behavior and Reproduction of Animals? *J Agric Environ Ethics* (2018) 31:197–218. doi:10.1007/s10806-018-9718-7
43.
Yates D, Leedham R. Prepubertal neutering of dogs — some risks and benefits. *Companion Animal* (2019) 24:38–42. doi:10.12968/coan.2019.24.1.38
44.
Kass PH, Johnson KL, Weng H-Y. Evaluation of animal control measures on pet demographics in Santa Clara County, California, 1993-2006. *PeerJ* (2013) 1:e18. doi:10.7717/peerj.18
45.
Adams V, Walker S, Taylor C. Attitudes to and opinions of neutering in dogs: results of a canine reproduction survey of veterinary surgeons. (2016)475–476.
46.

Miles CR, Bell CM, Pinkerton ME, Soukup JW. Maxillary ameloblastic fibroma in a dog. *Vet Pathol* (2011) 48:823–826. doi:10.1177/0300985810382091
47.
Cocia RI, Rusu AS. Attitudes of Romanian Pet Caretakers towards Sterilization of Their Animals: Gender Conflict Over Male, but Not Female, Companion Animals. *Anthrozoös* (2010) 23:185–191. doi:10.2752/175303710X12682332910097
48.
Houpt KA, Goodwin D, Uchida Y, Baranyiová E, Fatjó J, Kakuma Y. Proceedings of a workshop to identify dog welfare issues in the US, Japan, Czech Republic, Spain and the UK. *Appl Anim Behav Sci* (2007) 106:221–233. doi:10.1016/j.applanim.2007.01.005
49.
Hart BL. Effect of gonadectomy on subsequent development of age-related cognitive impairment in dogs. *J Am Vet Med Assoc* (2001) 219:51–56. doi:10.2460/javma.2001.219.51
50.
Faver CA. Sterilization of companion animals: exploring the attitudes and behaviors of Latino students in south Texas. *J Appl Anim Welf Sci* (2009) 12:314–330. doi:10.1080/10888700903163534
51.
Villaverde Haro C. Canine and feline obesity - tackling a growing problem. *Veterinary Times* (2015) 45:12, 14.
52.
Bushby PA, Griffin B. An overview of pediatric spay and neuter benefits and techniques. *Veterinary Medicine* (2011) 106:83–86, 88–89.
53.
Fielding WJ. Changing attitudes and animal welfare in small island developing states: dogs on new providence, the bahamas. *J Appl Anim Welf Sci* (2017) 20:65–74. doi:10.1080/10888705.2016.1240043
54.
Bednarczyk-Szurmak M, Bombik E, Bombik T, Łagowska K, Szumigłowska I, Różewicz M. Fighting homelessness of dogs - evaluation of the strategy adapted by the city of Siedlce. *Acta Scientiarum Polonorum - Zootechnica* (2015) 14:25–32.
55.
Looney AL, Bohling MW, Bushby PA, Howe LM, Griffin B, Levy JK, Eddlestone SM, Weedon JR, Appel LD, Rigdon-Brestle YK, et al. The Association of Shelter Veterinarians veterinary medical care guidelines for spay-neuter programs. *J Am Vet Med Assoc* (2008) 233:74–86. doi:10.2460/javma.233.1.74
56.
Ponglowhapan S, Church DB, Khalid M. Differences in the proportion of collagen and muscle in the canine lower urinary tract with regard to gonadal status and gender. *Theriogenology* (2008) 70:1516–1524. doi:10.1016/j.theriogenology.2008.06.099
57.
Lima LR de, Fontana CD, Eda HM, Ribeiro RM, Faleiros RR. Low-cost neutering program and its postsurgical complications for dogs and cats. *International Journal of Veterinary Science* (2016) 5:122–126.
58.
Muraro L, White RS. Complications of ovariohysterectomy procedures performed in 1880 dogs. *Tierarztl Prax Ausg K Klientiere Heimtiere* (2014) 42:297–302.
59.
Fielding WJ, Gall M, Green D, Eller WS. Care of dogs and attitudes of dog owners in Port-au-Prince, the Republic of Haiti. *J Appl Anim Welf Sci* (2012) 15:236–253. doi:10.1080/10888705.2012.683760
60.
Mongillo P, Scandurra A, D'Aniello B, Marinelli L. Effect of sex and gonadectomy on dogs' spatial performance. *Appl Anim Behav Sci* (2017) 191:84–89. doi:10.1016/j.applanim.2017.01.017
61.
McKay SA, Farnworth MJ, Waran NK. Current attitudes toward, and incidence of, sterilization of cats and dogs by caregivers (owners) in Auckland, New Zealand. *J Appl Anim Welf Sci* (2009) 12:331–344. doi:10.1080/10888700903163617
62.
Dolan ED, Weiss E, Slater MR. Welfare Impacts of Spay/Neuter-Focused Outreach on Companion Animals in New York City Public Housing. *J Appl Anim Welf Sci* (2017) 20:257–272. doi:10.1080/10888705.2017.1305904
63.
Hoad JG. Spaying bitches: why, when, how? *Vet Nurs* (2018) 9:418–421. doi:10.12968/vetn.2018.9.8.418
64.
Mogheiseh A, Nikahval B, Ahmadi N, Yazdanpanah R, Sadat Z, Nazifi S. Bilateral ovarian pedicle ligation as an alternative to ovariectomy and ovarian response to eCG treatment. *Comp Clin Path* (2017) 26:197–202. doi:10.1007/s00580-016-2369-z

65.
D'Onise K, Hazel S, Caraguel C. Mandatory desexing of dogs: one step in the right direction to reduce the risk of dog bite? A systematic review. *Inj Prev* (2017) 23:212–218. doi:10.1136/injuryprev-2016-042141
66.
Perez-Marin CC, Molina L, Vizuete G, Sanchez JM, Zafra R, Bautista MJ. Uterine and ovarian remnants in an incorrectly spayed bitch: a case report. *Veterinarni Medicina* (2014) 59:102–106. doi:10.17221/7320-VETMED
67.
Sones E. Neoplastic considerations for spaying and neutering dogs. *Clinical Theriogenology* (2019) 11:239–242.
68.
Garcia RCM, Amaku M, Biondo AW, Ferreira F. Dog and cat population dynamics in an urban area: evaluation of a birth control strategy. *Pesq Vet Bras* (2018) 38:511–518. doi:10.1590/1678-5150-pvb-4205
69.
McIntyre RL, Levy JK, Roberts JF, Reep RL. Developmental uterine anomalies in cats and dogs undergoing elective ovariohysterectomy. *J Am Vet Med Assoc* (2010) 237:542–546. doi:10.2460/javma.237.5.542
70.
McKenzie B. Evaluating the benefits and risks of neutering dogs and cats. *CAB Reviews* (2010) 5: doi:10.1079/PAVSNNR20105045
71.
Valenta K, Gettinger-Larson JA, Chapman CA, Farris ZJ. Barking up the right tree: Understanding local attitudes towards dogs in villages surrounding Ranomafana National Park, Madagascar can benefit applied conservation. *MCD* (2016) 11:87. doi:10.4314/mcd.v11i2.4
72.
Pelander L, Hagman R, Häggström J. Concentrations of cardiac Troponin I before and after ovariohysterectomy in 46 female dogs with pyometra. *Acta Vet Scand* (2008) 50:35. doi:10.1186/1751-0147-50-35
73.
Scandurra A, Alterisio A, Di Cosmo A, D'Ambrosio A, D'Aniello B. Ovariectomy Impairs Socio-Cognitive Functions in Dogs. *Animals (Basel)* (2019) 9: doi:10.3390/ani9020058
74.
Filipenco N, Baraitareanu S. Assessment of owner's perception concerning role of neutering and spaying in welfare of dogs. *Scientific Works - University of Agronomical Sciences and Veterinary Medicine, Bucharest Series C, Veterinary Medicine* (2012) 58:277–284.
75.
Mogheiseh A, Nikahval B, Ahrari Khafi MS, Mansourian M, Nazifi S, Mardani Z. Effects of bilateral whole vessel ovarian ligation on dogs' ovarian function and histopathology. *Comp Clin Path* (2018) 27:1085–1091. doi:10.1007/s00580-018-2705-6
76.
de Bleser B, Brodbelt DC, Gregory NG, Martinez TA. The association between acquired urinary sphincter mechanism incompetence in bitches and early spaying: a case-control study. *Vet J* (2011) 187:42–47. doi:10.1016/j.tvjl.2009.11.004
77.
Reichler IM, Welle M, Sattler U, Jöchle W, Roos M, Hubler M, Barth A, Arnold S. Comparative quantitative assessment of GnRH- and LH-receptor mRNA expression in the urinary tract of sexually intact and spayed female dogs. *Theriogenology* (2007) 67:1134–1142. doi:10.1016/j.theriogenology.2007.01.001
78.
Brent L. Growing interest in hormone sparing dog sterilization and recommendations for standard identification methods. *Clinical Theriogenology* (2019) 11:247–253.
79.
Szczubial M, Kankofer M, Bochniarz M, Dąbrowski R. Effects of ovariohysterectomy on oxidative stress markers in female dogs. *Reprod Domest Anim* (2015) 50:393–399. doi:10.1111/rda.12501
80.
Forsee KM, Davis GJ, Mouat EE, Salmeri KR, Bastian RP. Evaluation of the prevalence of urinary incontinence in spayed female dogs: 566 cases (2003-2008). *J Am Vet Med Assoc* (2013) 242:959–962. doi:10.2460/javma.242.7.959
81.
Bailey CS. Non-cancerous conditions associated with spay/neuter status in the canine. *Clinical Theriogenology* (2016) 8:203–206.
82.
Howe L. Current perspectives on the optimal age to spay/castrate dogs and cats. *VMRR* (2015)171. doi:10.2147/VMRR.S53264
83.

Hart B, Hart L, Thigpen A, Willits N. Best age for spay and neuter: a new paradigm. *Clinical Theriogenology* (2019) 11:235–237.
84.
Balogh O, Borruat N, Andrea Meier A, Hartnack S, Reichler IM. The influence of spaying and its timing relative to the onset of puberty on urinary and general behaviour in Labrador Retrievers. *Reprod Domest Anim* (2018) 53:1184–1190. doi:10.1111/rda.13225
